# Supplementary material for: Predicting multiple sclerosis from radiologically isolated syndrome using generative artificial intelligence
Source: PLOS Digit Health. 2026 Apr 24;5(4):e0001374. doi: 10.1371/journal.pdig.0001374 (PMC13108761; doi:10.1371/journal.pdig.0001374)
Supplement: Attachment — Submitted filename: 2026 response to reviewer Plos D.docx [file pdig.0001374.s002.docx]

Nice, February 19, 2026

Dear Editor,

Thank you for granting us the opportunity to revise our paper further. We have made sincere efforts to address the requirements outlined by the editorial team and reviewers. We have revised the paper to address all points. Below we provide a point-by-point response in blue, with

reviewer comments in italics. All manuscript changes are indicated in tracked changes in the revised file.

We are optimistic that, with these modifications, our manuscript is now well-suited for publication in PLOS Digital Health.

**Journal Requirements:**

*We do not publish any copyright or trademark symbols that usually accompany proprietary names, eg (R), (C), or TM  (e.g. next to drug or reagent names). Please remove all instances of trademark/copyright symbols throughout the text, including BrainGML®-MS.*

We thank the Editor for this reminder. In the revised version, we removed all trademarks and copyrights.

**Additional Editor Comments:**

*Clinical and methodological characterization: Reviewers requested additional clarity on the clinical features of your cohort, including whether converters developed RRMS versus PPMS, the potential impact of mixed field strengths (1.5T and 3T) on volumetric measurements, and whether cardiometabolic comorbidities—which could independently influence brain age gap—were documented. Please also confirm explicitly that RIS individuals met the 2023 diagnostic criteria.*

We thank the reviewers for raising these significant points. We detail below how and where we addressed each point regarding phenotypes, mixed fields, and considerations for comorbidities.

*Generalizability and clinical utility: A central concern is that the model has not been validated on an independent cohort. Additionally, reviewers questioned whether the identified clusters provide meaningful risk stratification beyond established predictors (age, spinal cord lesions, CSF findings), given that the observed conversion rates overlap with previously published estimates. The discussion of clinical implementation would benefit from a more measured perspective on the distance between these findings and bedside application.*

We acknowledge that this is a major point that needs to be enhanced and clarified. We added and highlighted changes in the revised manuscript.

*Reproducibility and transparency. We note that while the underlying segmentation tool (AssemblyNet) is publicly available, the BrainGML-MS algorithm itself is proprietary. For a study whose primary contribution is a novel AI-driven prognostic tool, this presents a significant barrier to independent replication and external validation by other research groups. We ask the authors to address this limitation explicitly in the revised manuscript and, where possible, provide additional methodological detail sufficient to allow readers to evaluate the approach critically. If regulatory or commercial considerations preclude full code release, the authors should discuss what steps might enable future independent validation (e.g., collaborative access arrangements, validation APIs, or plans for eventual open-source release). Similarly, further explanations of the underlying mechanisms can be made even in the absence of full code release.*

We thank the editor and reviewers for raising this point. It is now clearly added and highlighted in the revised version.

**Reviewers' Comments:**

*Reviewer #1: This manuscript uses a new unsupervised AI-driven model (BrainGML-MS) to determine if factors could be found that could determine if persons with radiologically isolated syndrome/asymptomatic or presymptomatic multiple sclerosis (MS) were at higher risk of conversion to having clinical manifestations of MS. This is an important study as although risk factors have been identified for RIS converting to clinical MS, these are not definitive and so if other factors could be uncovered with a relatively unbiased model, if you will, it would be helpful. Volumetric differences were seen between RIS-C and RIS-NC with RIS-C having atrophy of structures more similar to MS, with additional impact of the presence of spinal cord lesions. One of the strengths of the study is inclusion of healthy controls and those already with clinical manifestations of MS as comparators to RIS. I have a few comments seeking clarification for certain aspects of the manuscript.*

We deeply appreciate the reviewer's nice comment on our manuscript.

*Major Comments:
1. Did all the RIS-C participants develop RRMS or did some develop PPMS? If RIS-C participants developed RRMS and PPMS, was there anything additional in the model that was indicative of participants developing RRMS vs. PPMS? One would anticipate spinal cord lesions would be a factor given this has been previously shown by your RISC (Kantarci et al., Ann Neurol 2016), but this is already raised by your model in general for RIS-C vs. RIS-NC.*

We thank the reviewer for raising this important point. In all RIS-C patients, RRMS developed. As progression from RIS to PPMS is very rare, we did not have any to report in this series. Therefore, subtype-specific analyses comparing relapsing-remitting MS (RRMS) and PPMS were not possible. We have clarified this explicitly in the Results section to avoid ambiguity. We have removed the prediction of the clinical profile from the introduction (page 4). We specifically mentioned this in the revised version (page 5) and added it to the discussion section (page 13).

*2. Was there any impact of magnetic field strength on the results, given data acquired at both 1.5T and 3T was acquired? Was 1.5T felt to be sufficient for volumetrics with the model?*

We thank the reviewer for this important methodological remark. In our study, RIS and MS patient scans were all acquired using standardized clinical 3T protocols. This is now mentioned on page 14. The mixed-field dataset (1.5T and 3T) was applied only to the large external healthy reference database used to construct normative models and generate digital twins. Inclusion of multi-field-strength controls was intentional, as it improves model robustness by incorporating scanner-related variability into the normative distribution. This design reduces the risk that deviations detected in patients reflect scanner differences rather than biological variation. This has now been added to page 15. Furthermore, prior validation of the segmentation pipeline used here has shown reliable volumetric performance across field strengths after preprocessing steps, including intensity normalization, bias correction, and spatial standardization. These preprocessing procedures were applied uniformly to all images in our study. Regarding whether 1.5T is sufficient for volumetric analysis, existing literature and our validation experiments indicate that volumetric measures derived from T1-weighted MRI can be reliably estimated at 1.5T when standardized preprocessing is applied. We have clarified this in the methods (page 25). We chose AssemblyNet for brain segmentation in this study because it has been shown to be robust to acquisition variability across scanners. Two recent evaluations published in *Scientific Reports*demonstrated that, among multiple segmentation algorithms compared (AssemblyNet, FastSurfer, FreeSurfer), AssemblyNet exhibited the lowest errors and most consistent volumetric estimates across 1.5 T and 3 T data, indicating reduced sensitivity to magnetic field strength variability and other machine effects in large multi-site datasets.

We also added 2 references (31,32):

Piot, E., Renard, F., Attyé, A. *et al.* Estimation of reference curves for brain atrophy and analysis of robustness to machine effects. *Sci Rep* **15**, 34585 (2025).

Bürkle, E., Nazzal, A., Debolski, A., Ernemann, U., Lindig, T., and Bender, B . Scan-rescan reliability assessment of brain volumetric analysis across scanners and software solutions. Sci Rep,15(1):29843 (2025).

*3. Is there any data on cardiometabolic comorbidities (hypertension, hyperlipidemia, type 2 diabetes, smoking) for the participants? This could influence the brain age gap and so if this information is available, would be helpful to include given that differences between many group comparisons were significant as well. Cardiometabolic comorbidities are potentially reversible, so if present in the RIS-C group, could also be helpful to include as part of management to try to prevent conversion to MS.*

We thank the reviewer for this insightful suggestion. We agree that cardiometabolic and lifestyle factors such as diabetes, smoking, hypertension, and obesity can influence brain structure and may affect brain age estimates. These variables are therefore important potential confounders when interpreting structural imaging biomarkers. Unfortunately, systematic data on cardiometabolic comorbidities were not available for all participants in the retrospective datasets used in this study. Because these variables were inconsistently recorded across sites and time points, we were unable to include them reliably in the current analyses without introducing selection bias. We have clarified this limitation in the methods (page 15) and in the limitations (page 13). We also agree with the reviewer that such variables could be clinically relevant if shown to modify risk. We have therefore tempered our interpretation and expanded the discussion of potential clinical implications (page 14).

*Presumably all RIS individuals met 2023 RIS criteria? If so, this could be stated in the Patient Selection section of the Materials and Methods.*

We thank the reviewer for noticing the missing information. This has now been added to the revised version on page 16.

*Reviewer #2: In this paper the Authors evaluated whether a new unsupervised AI-driven model could stratify individuals with RIS at a greater risk of conversion. A total of 152 RIS individuals that will evolve to clinical MS or not during the follow-up were compared to 152 MS patients and 152 healthy controls. The unsupervised analysis identified four RIS clusters with varying conversion risks, ranging from 10% to 39% over five years. Brain aging patterns and the presence of spinal cord lesions differed significantly among groups.
The approach taken in this paper is of clear scientific interest and novelty.*

We sincerely thank the reviewer for their careful evaluation and for recognizing the scientific interest and novelty of our work. We address each comment below and have revised the manuscript accordingly.

*I found, however, the following issues:
-- a concern about generalizability of the results, as the proposed clustering and risk stratification are not validated on an independent cohort of RIS subjects;*

We agree that external validation is an essential step for confirming generalizability. The present study was designed as a methodological and proof-of-concept investigation using the largest available RIS cohort with standardized MRI and clinical follow-up. Because RIS is a rare condition, independent cohorts with comparable imaging protocols and longitudinal data remain limited. Importantly, our framework does not rely on cohort-specific parameter fitting; instead, it uses individualized normative modeling based on a large external healthy reference dataset (>4,000 subjects across 103 sites). This reduces the risk of overfitting and enhances robustness to dataset heterogeneity. To address this concern, we have explicitly clarified the limitation in the Discussion, added a paragraph describing planned external validation, and tempered claims about generalizability (page 13).

*-- to my knowledge the BrainGML®-MS algorithm is not publicly available, which is an obstacle to independent replication of the data or assessment of its methodological characteristics.*

We fully agree that transparency and reproducibility are critical. The software includes proprietary components that are currently undergoing regulatory certification as medical devices, which restricts the public release of the full code. However, we have clarified that the methodological pipeline is sufficiently detailed for replication, including preprocessing, segmentation, and dimensionality-reduction steps that rely on publicly available methods. Derived data and parameter specifications can be shared upon reasonable request. We have revised the manuscript to explicitly address reproducibility (page 24). While the full BrainGML-MS software cannot be publicly released due to regulatory and proprietary constraints, we have made substantial efforts to ensure transparency and methodological reproducibility.

First, we have published the complete methodological protocol on [protocols.io](http://protocols.io) (DOI provided in the manuscript and private link for reviewers:

<https://www.protocols.io/private/B2FD26720C5011F1ABCD0A58A9FEAC02> to be removed before publication), where all preprocessing steps, feature extraction procedures, manifold learning strategies, clustering methods, and statistical analyses are described in detail. This ensures that independent researchers can replicate the analytical workflow.

Second, we have made available a dedicated GitHub repository (<https://github.com/Geodaisics-code/ScoRIS>) containing the reduced manifold-space coordinates, clustering assignments, and associated clinical variables used in the study. By sharing these derived data, we enable independent validation of our statistical analyses, replication of clustering results, and further methodological benchmarking.

Although the proprietary implementation of BrainGML-MS itself cannot be distributed, the combination of a fully documented protocol and openly shared derived datasets reflects our commitment to reproducibility, transparency, and scientific rigor.

We hope this effort adequately addresses concerns regarding accessibility and independent validation.

*-- it remains insufficiently demonstrated whether BAG and cluster outperform or add to other already known strong predictors of evolution (e.g., age, spinal cord lesions, CSF OCBs…).*

We thank the reviewer for this important point. Our primary objective was to determine whether unsupervised imaging-derived structure could identify biologically meaningful subgroups, rather than to build a supervised prediction model. Therefore, the study was not originally powered to formally compare predictive performance against existing clinical predictors. However, we agree that demonstrating incremental value strengthens clinical relevance. To address this, we clarified the study aim (phenotypic stratification rather than replacing the prediction model). We added analyses evaluating associations between cluster membership and known predictors (page 8). We revised the Discussion to frame BAG as a complementary biomarker rather than a replacement (page 13).

*-- conversion probabilities (10–39% at 5 years) overlap substantially with previously reported RIS risks, Therefore, does the model really provide clinically useful stratification beyond existing risk models?.*

We appreciate this insightful observation. The key value of our model is not altering the global conversion rate but partitioning individuals into subgroups with different risk profiles within that range. Traditional estimates provide population-level risk, whereas our approach provides individualized structural phenotypes. We have clarified this distinction in the manuscript (page 13).

*--While the discussion suggests implications for early treatment decisions, clinical implementation appears rather speculative. This issue should be discussed using a more critical approach.*

We thank the reviewer for this important comment. We agree and have revised the Discussion to moderate claims regarding clinical translation. The manuscript now emphasizes that clinical implementation requires prospective validation, health-economic evaluation, and regulatory approval. We removed speculative language and replaced it with cautious interpretation (page 14).

**Figure resubmission:**

*While revising your submission, we strongly recommend that you use PLOS’s NAAS tool (*[*https://ngplosjournals.pagemajik.ai/artanalysis*](https://ngplosjournals.pagemajik.ai/artanalysis)*) to test your figure files. NAAS can convert your figure files to the TIFF file type and meet basic requirements (such as print size, resolution), or provide you with a report on issues that do not meet our requirements and that NAAS cannot fix.*

We thank the editor for sharing the PLOS’s NAAS tool.  NAAS has confirmed that the figure files meet PLOS requirements, and we included these NAAS-processed figure files when submitting our revised manuscript.

**Reproducibility:**

*To enhance the reproducibility of your results, we recommend that authors of applicable studies deposit laboratory protocols in* [*protocols.io*](http://protocols.io/)*, where a protocol can be assigned its own identifier (DOI) such that it can be cited independently in the future. Additionally, PLOS ONE offers an option to publish peer-reviewed clinical study protocols. Read more information on sharing protocols at* [*https://plos.org/protocols?utm_medium=editorial-email&utm_source=authorletters&utm_campaign=protocols*](https://plos.org/protocols?utm_medium=editorial-email&utm_source=authorletters&utm_campaign=protocols)

We thank the reviewers and the editor for their comments regarding code availability and reproducibility. While the full BrainGML-MS software cannot be publicly released due to regulatory and proprietary constraints, we have made substantial efforts to ensure transparency and methodological reproducibility.

First, we have published the complete methodological protocol on [protocols.io](http://protocols.io) (DOI provided in the manuscript; private link for reviewers): <https://www.protocols.io/private/B2FD26720C5011F1ABCD0A58A9FEAC02> to be removed before publication), where all preprocessing steps, feature extraction procedures, manifold learning strategies, clustering methods, and statistical analyses are described in detail. This ensures that independent researchers can replicate the analytical workflow. It is added on page 25.

Second, we have made available a dedicated GitHub repository (<https://github.com/Geodaisics-code/ScoRIS>) containing the reduced manifold-space coordinates, clustering assignments, and associated clinical variables used in the study. By sharing these derived data, we enable independent validation of our statistical analyses, replication of clustering results, and further methodological benchmarking.

Although the proprietary implementation of BrainGML-MS itself cannot be distributed, the combination of a fully documented protocol and openly shared derived datasets reflects our commitment to reproducibility, transparency, and scientific rigor. This is added on page 25.

We hope this effort adequately addresses concerns regarding accessibility and independent validation.
